# Integrated Analysis of Machine Learning and Deep Learning in Silkworm Pupae (*Bombyx mori*) Species and Sex Identification

**DOI:** 10.3390/ani13233612

**Published:** 2023-11-22

**Authors:** Haibo He, Shiping Zhu, Lunfu Shen, Xuening Chang, Yichen Wang, Di Zeng, Benhua Xiong, Fangyin Dai, Tianfu Zhao

**Affiliations:** 1 State Key Laboratory of Resource Insects, College of Sericulture, Textile and Biomass Sciences, Southwest University, Chongqing 400715, China; hhb_swu@126.com (H.H.);; 2 Chongqing Engineering Research Center of Biomaterial Fiber and Modern Textile, Chongqing 400715, China; 3 College of Engineering and Technology, Southwest University, Chongqing 400715, China; zspswu@126.com; 4 Yibin Academy of Southwest University, Yibin 644000, China; 5 Institute of Sericulture Science and Technology Research, Chongqing 400700, China

**Keywords:** silkworm pupae, species and sex identification, feature extraction, global modeling, machine learning, deep learning

## Abstract

**Simple Summary:**

Identifying silkworm pupae species and sex accurately is essential for the hybrid pairing of corresponding species in sericulture, which guarantees the quality of the silkworm eggs and silk. However, there is no cost-effective method that offers a labor-saving and intelligent solution for this. In this study, machine learning and deep learning are used for the automatic recognition of pupae species and sex, either separately or simultaneously, based on the patterns perceived from images. A vast number of postural images of pupae were used for global modeling to eliminate the impact of posture on recognition rate. Six traditional descriptors and six deep learning descriptors were employed for feature extraction and then combined with three machine learning classifiers for identification. Based on that, the model with the best identification performance was screened out, and it can serve as a reference for sericulture breeding.

**Abstract:**

Hybrid pairing of the corresponding silkworm species is a pivotal link in sericulture, ensuring egg quality and directly influencing silk quantity and quality. Considering the potential of image recognition and the impact of varying pupal postures, this study used machine learning and deep learning for global modeling to identify pupae species and sex separately or simultaneously. The performance of traditional feature-based approaches, deep learning feature-based approaches, and their fusion approaches were compared. First, 3600 images of the back, abdomen, and side postures of 5 species of male and female pupae were captured. Next, six traditional descriptors, including the histogram of oriented gradients (HOG), and six deep learning descriptors, including ConvNeXt-S, were utilized to extract significant species and sex features. Finally, classification models were constructed using the multilayer perceptron (MLP), support vector machine, and random forest. The results indicate that the {HOG + ConvNeXt-S + MLP} model excelled, achieving 99.09% accuracy for separate species and sex recognition and 98.40% for simultaneous recognition, with precision–recall and receiver operating characteristic curves ranging from 0.984 to 1.0 and 0.996 to 1.0, respectively. In conclusion, it can capture subtle distinctions between pupal species and sexes and shows promise for extensive application in sericulture.

## 1. Introduction

Being the birthplace of global sericulture and the foremost silk producer and exporter, China has boosted rural revitalization and sericulturists’ earnings. For example, sericulturists in Guangxi Province, China, earned CNY 20.818 billion from cocoon sales in 2021 [1]. In addition, sericulture impacts the global economy and culture considerably. Sorting silkworm pupae sexes affects the hybridization rate of eggs and the market competitiveness of silk [2]. However, various factors, such as production seasonality, brief pupal duration, and rising labor costs, impose challenges in the sorting process [3].

Since the last century, researchers have been developing nondestructive and reliable sex sorting methods. Initially, biologists developed silkworm species with sex-linked traits to ease pupal sex sorting [4,5]; however, these represent only a small fraction of the species. DNA and amino acid analyses can determine sex but are destructive [6,7]. Recently, powerful spectral and visual techniques have been proposed to identify pupal sexes. These techniques include magnetic resonance imaging (MRI) [8], X-ray imaging [9], hyper-spectral imaging (HSI) [10,11], near-infrared (NIR) spectroscopy [12], and image recognition [13]. Among these, NIR is frequently used for sex determination based on differences in morphology, gonadal traits such as eggs, and water content between male and female pupae [14,15,16,17,18]. Having addressed sericulture challenges in areas like Shandong, China, Zhu et al. developed an NIR system with 97.5% accuracy and a sorting rate of 7.7 pupae per second [19]. However, NIR requires frequent manual modeling, and the relatively high cost of its components limits its applicability.

Leveraging the stable genetic traits of pupae, low-cost image recognition has shown significant promise in sex identification. Kamtongdee et al. and Tao et al. identified pupa sex based on gonadal traits through image analysis [20,21,22,23]. However, these methods require precise pupa positioning, making them less suitable for production lines. Note that methods using pupal appearance can overcome this drawback. Liang et al. achieved a 98% sex recognition rate using machine learning [24], and Yu et al. obtained 97% accuracy using deep learning [25]. However, their studies must address the impact of varying pupae posture on feature extraction, and large datasets are required to train the model to ensure practical feasibility.

As the field advances, research on pupae species identification has been conducted alongside studies on sex identification [26,27]. Identifying pupal species provides an objective basis for silkworm identity, thereby reducing species mix-ups in breeding and ensuring correct hybridization of the corresponding species. However, there is no reliable and cost-effective method that offers an intelligent and labor-saving solution for this. In response, this study combines image recognition with machine learning and deep learning techniques to develop a model to identify pupae species and sex, either separately or simultaneously, to meet the needs of silkworm breeding factories. Through global modeling of the pupal back, abdomen, and side postures, this proposed method effectively addresses interference caused by changing pupal positions [19,28].

**Figure 1:**
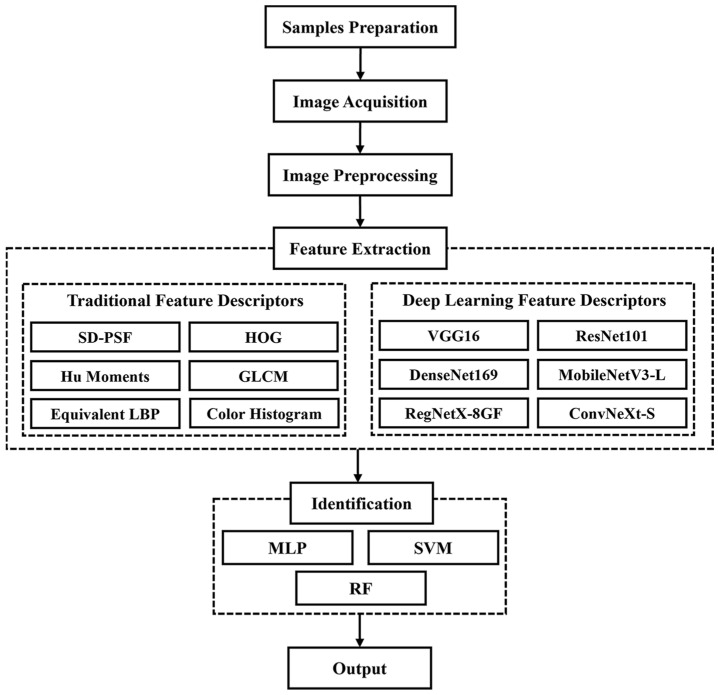
Flowchart of the identification of silkworm pupae species and sex.

Figure 1 shows a flowchart of the proposed method. First, images of pupae from both sexes across five species were captured. Then, six traditional descriptors (self-designed pupal shape feature (SD-PSF), Hu moments, histogram of oriented gradients (HOG), equivalent local binary pattern (LBP), gray-level co-occurrence matrix (GLCM), and color histogram) and six deep learning descriptors (VGG16, ResNet101, DenseNet169, MobileNetV3-L, RegNetX-8GF, and ConvNeXt-S) were used to extract features from pupae images. The extracted features were then input to three classifiers, namely multilayer perceptron (MLP), support vector machine (SVM), and random forest (RF), to perform the recognition task.

In summary, this paper makes the following contributions:

(1) An economical and intelligent solution for sericulture breeding has been proposed.
(2) A global modeling approach was proposed that, using the constructed pupae image set, addressed posture-related identification challenges.
(3) The {HOG + ConvNeXt-S + MLP} model with the top recognition rate was screened based on an integrated analysis.
(4) The advantages and disadvantages of the proposed model were compared with other techniques.

## 2. Materials and Methods

### 2.1. Sample Preparation

Live pupae from five species bred in autumn 2022 were sourced from the Institute of Sericulture Science and Technology Research in Chongqing, China, including the 7532 and 872 species from the Japanese system and the 871, FuRong, and HaoYue species from the Chinese system. Furthermore, skilled workers selected 120 female and 120 male pupae from each species based on the gonadal texture of their tails.

### 2.2. Image Acquisition and Data Partitioning

To capture pupae images, a system was configured using a digital single-lens reflex camera (D90, Nikon, Tokyo, Japan), paired with a zoom lens (AF-S NIKKOR 18–105 mm f/3.5–5.6 G ED, Nikon, Tokyo, Japan) mounted on a tripod (CVT–999RM, YunTeng, Zhongshan, China). The shooting distance of the lens to the pupa was 34 cm. Under indirect natural light, images were acquired with an F5.6 aperture, sensitivity of 100, automatic exposure, and white balance. The captured images were saved in JPEG format with a resolution of 2848 × 4288 pixels.

**Figure 2:**
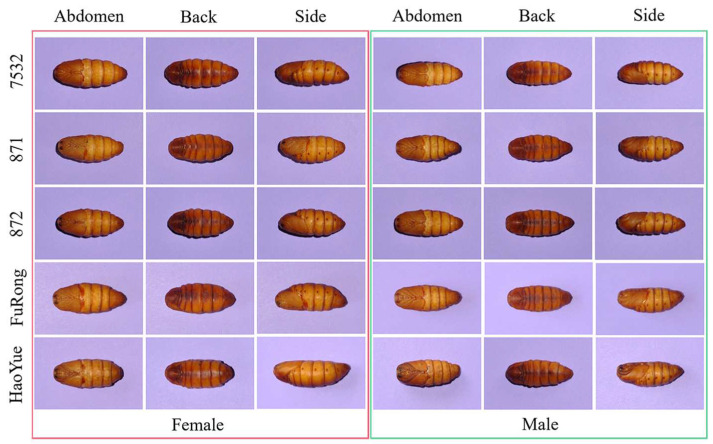
An example of back, abdomen, and side posture images of five species of male and female pupae. 7532, 871, 872, FuRong, HaoYue represent the five silkworm species.

Image acquisition for each species was conducted between the 9th and 11th days during the pupal stage. As shown in Figure 2, each pupa was imaged from the back, abdomen, and side views; therefore, a dataset of 3600 images was collected, representing male and female pupae from 5 species (10 classes with 360 images each). Given the significant differences in pupae weight by species and sex [13,29], weight served as the basis for data partitioning. For traditional approaches, the datasets were split at a ratio of 8:2 for training and testing. For deep learning and fusion approaches, the split was 8:1:1 for the training, calibration, and testing sets.

### 2.3. Image Preprocessing

**Figure 3:**
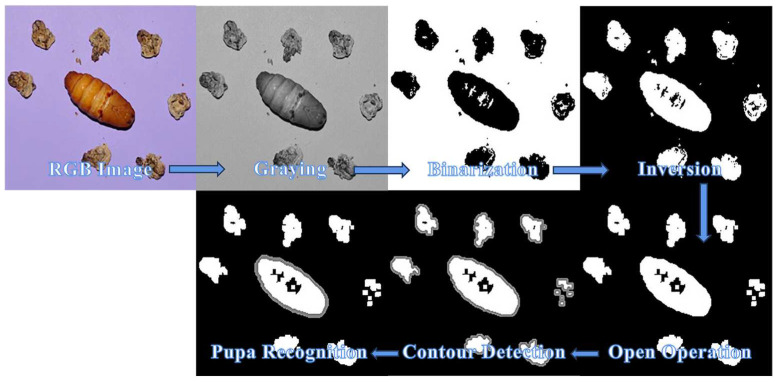
Pupa detection under strong interference.

To precisely separate the pupa from the background, the image preprocessing flow was based on sampled images and involved steps such as graying, binarization, image inversion, open operation, and contour detection. Figure 3 illustrates these operations, during which an area threshold was established for all identified contours to reduce interference from molts during image acquisition. After identifying the pupa, each image was cropped to 320 × 320 pixels (centered around the pupa mass) and then processed for feature extraction.

### 2.4. Feature Extraction

#### 2.4.1. Traditional Feature-Based Approaches

Features function as distinctive attributes for classifying input patterns. Traditional features are the basics of visual attributes, including shape, texture, color, and transform-based features [30]. Herein, six traditional descriptors were employed to extract features: SD-PSF, HOG, Hu moment, equivalent LBP, GLCM, and color histogram (Table 1).

**Figure 4:**
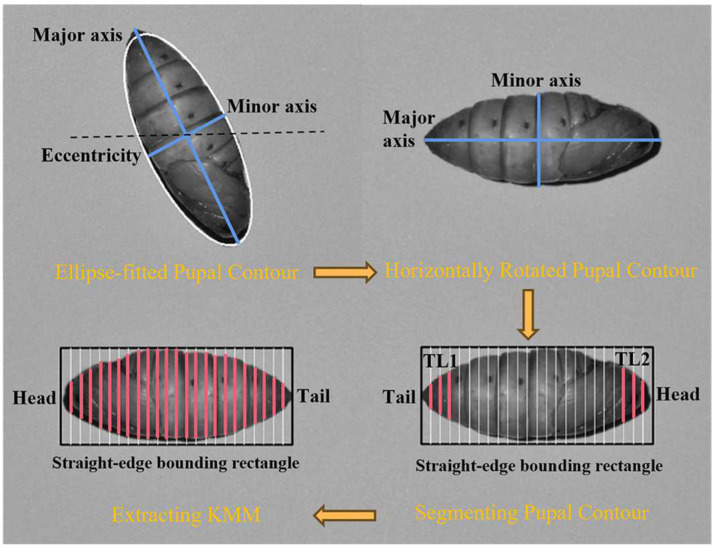
The process of extracting KMM. The black dashed line represents the horizontal line; the pink solid line and its length represent the KMM and its size.TL: total length.

The SD-PSF is derived from the pupal contour, and it includes parameters such as perimeter, area, and dispersity, as well as the minimum, average, and maximum radii, and the radius ratio. It also covers the major and minor axes, aspect ratio, rectangularity, circularity, and compactness by fitting the contour to shapes, e.g., rectangle, circle, and ellipse. In addition, the minor axis at K/24 of the pupal major axis (KMM, where K ranges from 1 to 23) represents the change in the contour’s curvature. As shown in Figure 4, the pupal contour can be placed horizontally using its major axis and the eccentricity of its minimum-fit ellipse. Furthermore, the KMM can be calculated using its straight-edge bounding rectangle. In addition, the pupal head and tail are determined by comparing TL1 and TL2. Herein, if TL2 > TL1, the pupal contour is flipped to maintain the order for feature extraction.

HOG is a descriptor characterizing an image’s local gradient direction and intensity, and is robust to illumination shifts and invariant to local geometric and photometric transformations [31]. Hu moments, geometrically invariant moments formed from second- and third-order central moments, excel in pattern recognition and image matching because of their robustness against zooming, translation, rotation, and mirroring [32].

GLCM, which is frequently employed in statistical texture analysis, extracts texture features from a gray-level co-occurrence matrix and captures an image’s details, including direction, interval, change range, and speed [10]. On the other hand, LBP operators highlight the local image texture structure and resist gray-scale variations [33]. Among them, the equivalent LBP takes advantage of numerical variations in the sequence of differential values between a central pixel and its surrounding pixels.

Color histograms serve as statistical representations of the frequency distribution of color intensity levels in images [30]. The color histogram, which decouples color information from grayscale and characterizes images based on hue, saturation, and intensity, is particularly well-suited for research in machine vision [34].

#### 2.4.2. Deep Learning Feature-Based Approaches

CNN can extract features directly from images through weight sharing and convolutional processes [35]. Table 2 describes the six CNN architectures explored in this study: VGG16 [36], ResNet101 [37], DenseNet169 [38], MobileNetV3-L [39], RegNetX-8GF [40], and ConvNeXt-S [41].

**Figure 5:**
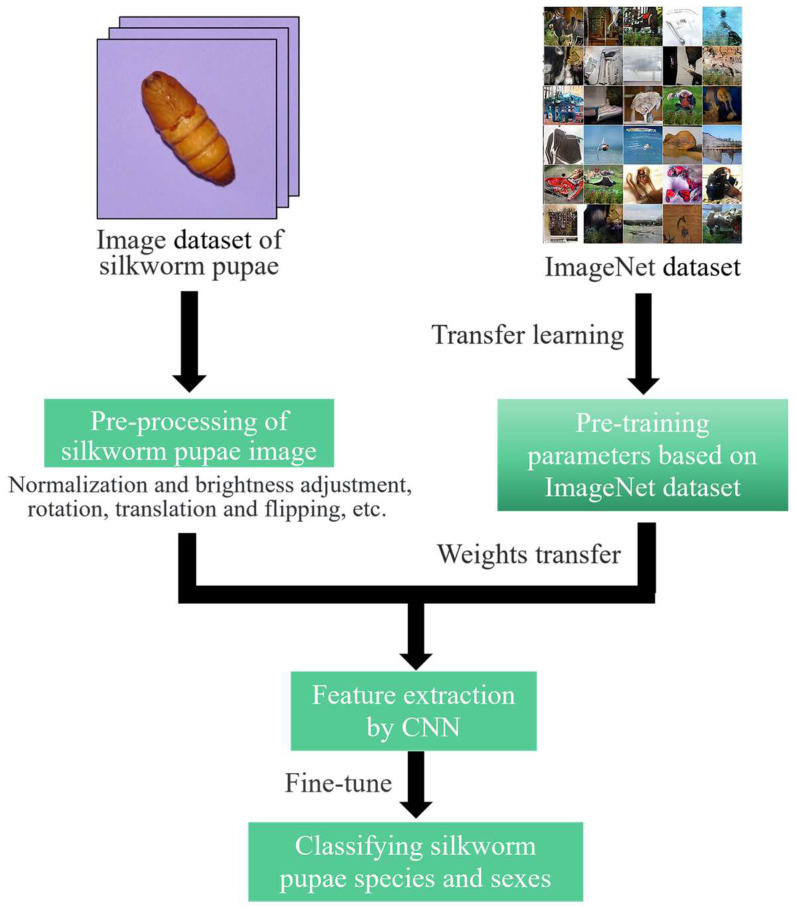
The research framework for deep learning feature-based approaches.

Figure 5 shows the research framework for the deep learning approaches. Initially, data augmentation was employed to enhance training performance and prevent model overfitting [42]. This process included the various transformations, such as flips, translations, rotations, and brightness adjustments, encountered when photographing the pupae. Transfer learning was used to expedite model convergence and boost accuracy [43]. Particularly, weights pretrained on ImageNet were used to initialize the CNN model, followed by fine-tuning at a lower learning rate (LR).

**Figure 6:**
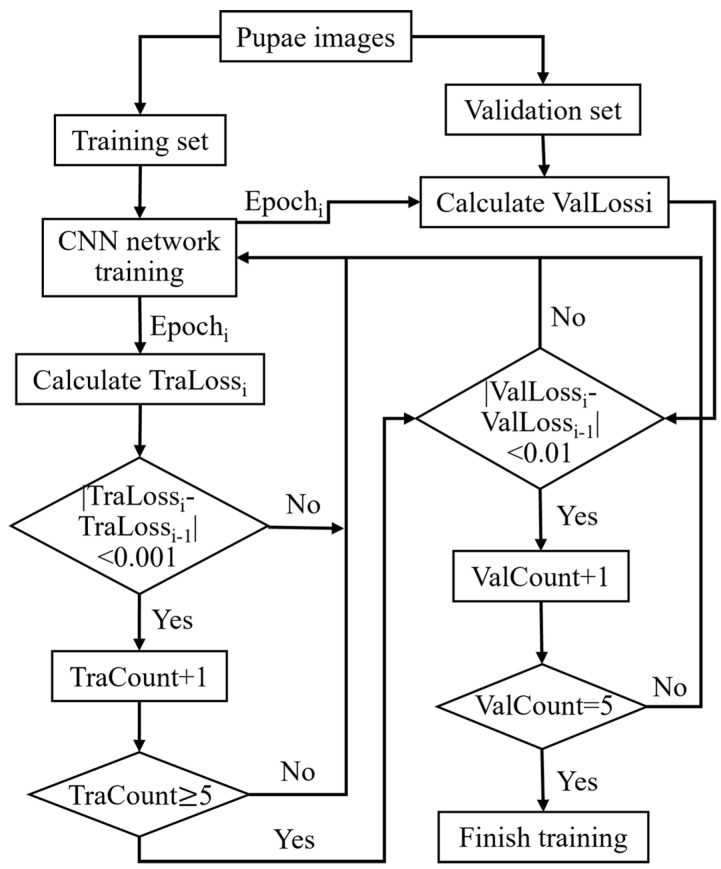
Flowchart of the early stopping method. Tra: train, Val: validation.

For both training and validation sets, the batch size was set to 64. During CNN training, stochastic gradient descent with a momentum of 0.9 and cross-entropy were employed as the optimizer and loss functions, respectively. A dynamic LR strategy was used, starting with an initial LR of 0.0001 and reducing it by a factor of 0.8 every five epochs of training. As shown in Figure 6, the early stopping method was employed to handle overfitting and halt training early. Herein, convergence began if training loss fluctuated by <0.001 over five epochs. If the validation loss shifts < 0.005 in another five epochs, it is considered fully converged, leading to early termination. Particularly, the best model parameters are saved from peak validation accuracy.

### 2.5. Identification

In this step, features extracted from the pupa image are input into a classifier for identification. The classifiers include MLP, SVM, and RF, and their parameters and settings are shown in Table 3. Herein, the traditional feature-based approach determines the optimal model hyperparameters through a grid search based on the highest accuracy of the classifier in five-fold cross-validation. These hyperparameters under consideration include the hidden layer size of the MLP, the penalty factor and kernel function’s penalty factor of the SVM, and the number of decision trees in the RF.

### 2.6. Performance Evaluation

The performance of the proposed model was evaluated based on accuracy, precision, recall, and F1-score. Identifying pupae species and sex involves multi-class classifications; therefore, the arithmetic mean of the metrics was used for all classes, as described in Equations (1)–(4).

(1) $$Accuracy=\frac{1}{n}\sum_{i=1}^{n}\frac{true\;positive+true\;negative}{true\;positive+ture\;negative+false\;positive+false\;negative}$$

(2) $$Precision=\frac{1}{n}\sum_{i=1}^{n}\frac{true\;positive}{true\;positive+false\;positive}$$

(3) $$Recall=\frac{1}{n}\sum_{i=1}^{n}\frac{true\;positive}{true\;positive+false\;negative}$$

(4) $$F1\;score=2\times \frac{Precision\times Recall}{Precision+Recall}$$

### 2.7. Hardware and Software

This study was executed on a workstation equipped with an Xeon^®^ Platinum 8353P @ 2.60 GHz processor (Intel, Santa Clara, CA, USA) and an GeForce RTX 3090 24 GB GPU (Nvidia, Santa Clara, CA, USA), and the primary programming language was Python.

## 3. Results and Discussion

### 3.1. Traditional Feature-Based Approaches

Table 4 shows the accuracy of the traditional descriptors for identifying pupae species and sex separately and simultaneously. Various descriptors, including singles and hybrids of two descriptors, were evaluated.

For the single descriptors, the MLP and SVM classifiers using {HOG} and RF using {Color Histogram} achieved peak accuracy rates of 88.47%, 89.44%, and 83.61% in {Species + Sex}, respectively. For species recognition, they reached 91.53%, 92.08%, and 87.92%, respectively. Conversely, {GLCM} was the least effective descriptor among all classifiers.

The hybrid of two descriptors was realized by concatenating the feature vectors of two single descriptors. Here, both MLP and SVM achieved 96.81% in species recognition using {HOG + Color Histogram} and 94.44% and 94.58%, respectively, for {Species + Sex} identification. Additionally, using {SD-PSF + Color Histogram}, the best recognition rates achieved by RF were 93.06% for species and 89.72% for {Species + Sex}. These results surpass the single descriptors, suggesting that the machine learning classifier can learn higher-dimensional vectors and achieve better classification. In contrast, {Hu Moments + GLCM} and {Equivalent LBP + GLCM} were the two least effective hybrid descriptors. Furthermore, {SD-PSF} and related descriptors excelled in sex recognition. Specifically, MLP and SVM with {SD-PSF + Equivalent LBP} and RF with {SD-PSF + Hu Moments} reached recognition rates of 97.78%, 97.50%, and 96.11%, respectively.

### 3.2. Deep Learning Feature-Based Approaches

Table 5 shows the classification accuracy of the deep learning descriptors in terms of pupae species and sexes.

For single descriptors, MLP, SVM, and RF with {ConvNeXt-S} achieved the highest accuracies for {Species, Sex, Species + Sex} at {97.20%, 96.97%, 95.28%}, {98.74%, 98.64%, 97.68%}, and {97.45%, 96.89%, 94.72%}, respectively. Thus, {ConvNeXt-S} was considered the best choice for the single descriptor. In contrast, {MobileNetV3-L} and {RegNetX-8GF} were the least effective descriptors among all classifiers.

For the hybrids of two descriptors that include {ConvNeXt-S}, MLP achieved the highest recognition rates for {Species, Sex, Species + Sex} using {ResNet101 + ConvNeXt-S} at {98.08%, 97.73%, 95.98%}, and the SVM reached {98.36%, 97.88%, 96.46%} with {DenseNet169 + ConvNeXt-S}. The RF obtained the highest recognition rates of {97.17%, 90.58%} for {Sex, Species + Sex} using {RegNetX-8GF + ConvNeXt-S}. However, these results show a decrease compared to those obtained using single descriptors. This indicates that deep learning performance relies not only on feature dimensions but also on the dataset scale and the interplay between descriptor structures. Moreover, descriptors linked to {MobileNetV3-L} were found to underperform in the classification tasks.

### 3.3. Fusion Approaches

Based on the above experimental results, this study selected the optimal traditional and deep learning descriptors from each classifier to conduct fusion experiments. Before the descriptors were concatenated, each descriptor’s feature dimensions were compressed to 500. As shown in Table 6, the MLP with {HOG + ConvNeXt-S} reached recognition rates of {99.09%, 99.09%, 98.40%} for {Species, Sex, Species + Sex}, while the RF with {Color Histogram + RegNet-8GF + ConvNeXt-S} obtained the lowest accuracy at {66.84%, 89.92%, 73.01%} for the same categories. Even though the accuracy of the {HOG + ResNet101 + ConvNeXt-S + MLP} model is lower compared to the {HOG + ConvNeXt-S + MLP} model, and the accuracy of the {HOG + DenseNet169 + ConvNeXt-S + SVM} model is lower compared to the {HOG + ConvNeXt-S + SVM} model, both can serve as references for more complex future classification tasks.

### 3.4. Comparison of Results by Different Feature Extraction Approaches

The experimental results in Table 4, Table 5 and Table 6 show that the {HOG + ConvNeXt-S + MLP} model achieved the top recognition rates of {99.09%, 99.09%, 98.40%} for {Species, Sex, Species + Sex}. This model outperformed the optimal traditional model {HOG + Color Histogram + SVM} by {2.28%, 2.15%, 3.82%} and the optimal deep learning model {ConvNeXt-S + SVM} by {0.35%, 0.45%, 0.72%}.

Table 7 compares the precision, recall, and F1-score of three models. For all models, precision outperforms recall, thereby suggesting fewer false positives. Based on these results, the features extracted by the deep learning approaches exhibit superior performance compared to the traditional approaches. For example, even when SVM uses {ConvNeXt-S}, its precision, recall, and F1 score (0.9772, 0.9767, and 0.9769) surpass the {HOG + Color Histogram} (0.9468, 0.9458, and 0.9463). In addition, the {HOG + ConvNeXt-S + MLP} model also achieved the highest precision, recall, and F1-score (0.9840, 0.9838, and 0.9839).

**Figure 7:**
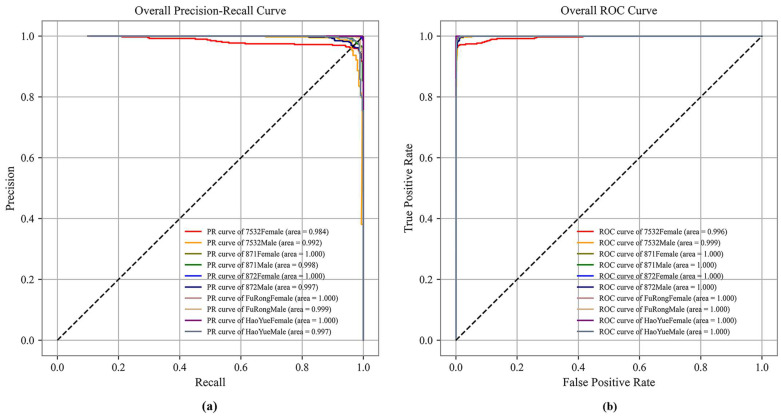
(**a**) PR curve and (**b**) ROC curve of the {HOG + ConvNeXt-S +MLP} model.

Figure 7 shows the precision–recall (PR) and receiver operating characteristic (ROC) curves to visualize model performance. The area under the PR and ROC curves for all classes in the {HOG + ConvNeXt-S + MLP} model ranges from 0.984 to 1.0 and 0.996 to 1.0, respectively. Here, the areas under the PR and ROC curves for 871Female, 872Female, FuRongFemale, and HaoYueFemale all reached 1.0. This reflects the high reliability of machine and deep learning methods for recognizing pupae species and sex through images.

**Figure 8:**
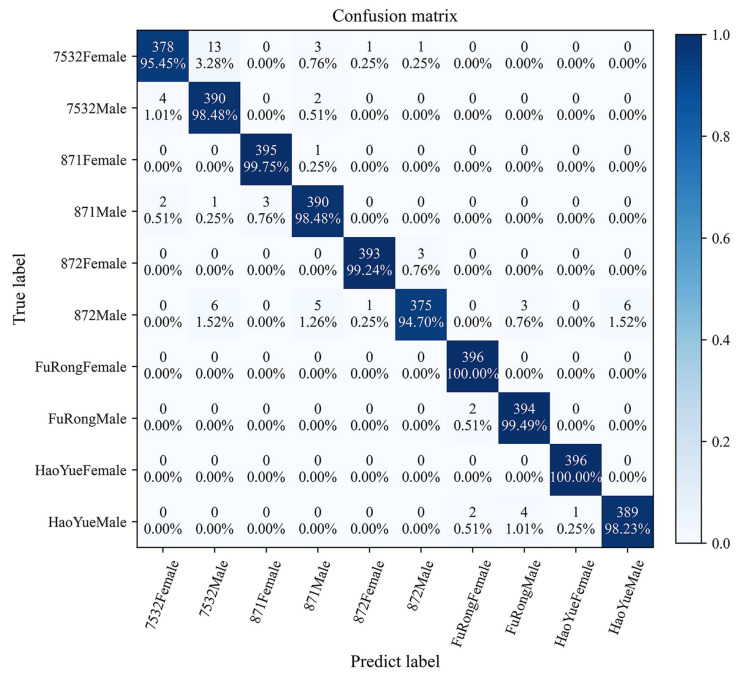
The confusion matrix of the {HOG + ConvNeXt-S +MLP} model.

Figure 8 further illustrates the more intuitive classification efficacy of the {HOG + ConvNeXt-S + MLP} model. The accuracy for all classes under this model ranges from 94.70% to 100%, with eight classes exceeding 98%. Particularly, the classification accuracies for the FuRongFemale and HaoYueFemale classes are 100%, corresponding to the RP and ROC curve results. In addition, misclassification mainly occurs between pupae of different sexes within the same species and those of the same sex across different species. On one hand, the {HOG + ConvNeXt-S + MLP} largely misclassifies 7532Male as 7532Female (and vice versa). On the other hand, it predominantly misclassifies 872Male as 7532Male, 871Male, FuRongMale, or HaoYueMale.

### 3.5. Comparison with Other Techniques

Compared to MRI [8], X-ray imaging [9], HSI [10], and NIR [19], the proposed global model has a lower cost; it is also nondestructive and nonradiative, unlike X-ray, DNA, and amino acid analysis [6,7]. The proposed model is based on stable genetic pupae traits unaffected by breeding conditions, thereby reducing the frequent modeling needs of NIR. It can also mitigate the effects of pupal postures on feature extraction and bypass precise positioning for methods based on the gonadal traits [21,23]. Furthermore, to the best of our knowledge, this is the first study to introduce global modeling to predict pupae species and sex, and it achieved 99.09% accuracy for separate identification and 98.40% for simultaneous recognition. Thus, the proposed global model is more suitable for practical application in sericulture.

## 4. Conclusions

Based on a dataset of posture images of silkworm pupae, this study aims to establish a global model to accurately identify the species and sex of pupae separately or simultaneously. Three machine learning classifiers (MLP, SVM, and RF) employed traditional descriptors (SD-PSF, HOG, Hu moments, equivalent LBP, GLCM, and color histogram) and deep learning descriptors (VGG16, ResNet101, DenseNet169, MobileNetV3-L, RegNetX-8GF, and ConvNeXt-S) to construct classification models. Next, the model performance of traditional, deep learning, and their fusion approaches were evaluated and compared. The findings demonstrate that the {HOG + ConvNeXt-S + MLP} model achieved top recognition rates of 99.09%, 99.09%, and 98.40% for species, sex, and species + sex, respecively. Additionally, its precision, recall, and F1-score were 0.9840, 0.9838, and 0.9839, with all classes having achieved an area under the PR and ROC curves exceeding 0.984 and 0.996, respectively. These results validate the effectiveness of machine learning and deep learning in recognizing the species and sexes of pupae through image analysis. Future research will collect a broader range of pupal images from different species and sexes to assess the proposed model’s generalizability under varied conditions. This will include the development of a finer-grained neural network based on HOG and ConvNeXt with the aim of improving the detection of subtle variations across species and sexes. Additional efforts will focus on modeling using datasets from diverse breeding batches to precisely identify stable hereditary phenotypic traits within individual pupal species.

## Figures and Tables

**Table 1:** Number of features extracted by six traditional descriptors and their descriptions.

Descriptors	Number of Features	Descriptions
SD-PSF	37	It includes features that measure pupal contour and curvature variations.
Hu moments	7	It includes seven combinations of second- and third-order central moments.
HOG	1296	Its parameters were set as 2 × 2 cells/block, 30 × 30 pixels/cell, and 9 histogram features/cell.
GLCM	20	After quantizing the image to 64 gray levels, calculations were performed at angles of 0°, 45°, 90°, and 135°. This was followed by computing angular moments, contrast, entropy, correlation, and inverse difference moments.
Equivalent LBP	59	It relies on numerical leaps in the ordering of differential values between the center pixel and its neighbors.
Color histogram	768	Hue, saturation, and intensity were each quantized to 256 dimensions.

**Table 2:** Details of the six CNN architectures.

Network	Depth	Size (MB)	Parameter (M)	Input	Output
VGG16	16	528	138	Normalized 224 × 224 RGB image	The 1000 class output for the ImageNet task serves as the extracted feature
ResNet101	101	171	44		
DenseNet169	169	55	14		
MobileNetV3-L	157	21	5		
RegNetX-8GF	22	151	5		
ConvNeXt-S	112	191	50		

**Table 3:** Details of parameter settings for each classifier.

Classifiers	Parameter Settings	
	Traditional Feature-Based Approaches	Deep Learning Feature-Based Approaches
MLP	Activation: Rectified Linear Unit, Solver: Limited-memory Broyden-Fletcher-Goldfarb-Shanno, Random State: 9	
	Hidden Layer Size∈{25, 50, 75, 100, 125, 150, (25, 25), (50, 50) (75, 75), (100, 100)}, Alpha∈{0.1, 0.01, 0.001, 0.0001, 0.00001, 0.000001}	Hidden Layer Size: 120, Alpha: 0.00001
SVM	Decision Function Shape: One-vs-Rest, Random State: 9	
	Kernel: Radial Basis Function, Degree: 3, C∈{2-5, 2-4, 2-3, 2-2, 2-1, 1, 21, 22, 23, 24, 25}, Gamma∈{2-15, 2-13, 2-11, 2-9, 2-7, 2-5, 2-3, 2-1, 21, 23, 25}	Kernel: Linear, C: 1
RF	Criteria: Gini Impurity, Minimum Samples for Split and Leaf: 2 and 1, Random state: 9	
	Number of Trees∈[200, 800](steps of 10)	Number of trees: 500

**Table 4:** Classification accuracy of traditional feature-based approaches.

Methods	Descriptors	Testing Accuracy {Species, Sex, Species + Sex} {%}		
		MLP	SVM	RF
Single descriptor	{SD-PSF}	{72.36, 94.44, 69.72}	{74.72, 95.14, 72.78}	{69.31, 95.14, 67.22}
	{HOG}	{91.53, 94.58, 88.47}	{92.08, 95.14, 89.44}	{78.19, 90.83, 74.03}
	{Hu Moments}	{73.75, 85.69, 65.97}	{72.78, 87.22, 65.28}	{75.42, 87.08, 68.33}
	{Equivalent LBP}	{80.56, 84.86, 74.03}	{81.39, 87.22, 76.11}	{71.67, 79.58, 64.44}
	{GLCM}	{63.61, 85.83, 58.89}	{67.92, 86.39, 63.47}	{58.33, 83.89, 52.22}
	{Color Histogram}	{89.17, 92.69, 85.00}	{90.41, 94.44, 87.22}	{87.92, 91.94, 83.61}
Hybrid of two descriptors	{SD-PSF + HOG}	{92.64, 95.69, 90.00}	{93.47, 96.53, 91.38}	{81.25, 92.08, 77.92}
	{SD-PSF + Hu Moments}	{89.17, 96.67, 86.67}	{89.72, 96.81, 87.22}	{87.08, 96.11, 84.31}
	{SD-PSF + Equivalent LBP}	{91.25, 97.78, 89.58}	{89.17, 97.50, 87.92}	{81.39, 95.28, 79.03}
	{SD-PSF + GLCM}	{86.11, 95.69, 83.75}	{84.72, 95.42, 82.22}	{78.33, 94.03, 76.25}
	{SD-PSF + Color Histogram}	{93.61, 95.00, 90.14}	{94.17, 95.83, 91.25}	{93.06, 95.14, 89.72}
	{HOG + Hu Moments}	{91.94, 95.42, 89.17}	{93.06, 95.42, 90.00}	{81.67, 91.53, 77.22}
	{HOG + Equivalent LBP}	{93.33, 96.53, 91.25}	{93.61, 96.39, 91.25}	{79.03, 90.97, 75.14}
	{HOG + GLCM}	{92.64, 95.27, 89.72}	{93.33, 95.00, 90.14}	{80.14, 91.39, 75.69}
	{HOG + Color Histogram}	{96.81, 96.94, 94.44}	{96.81, 96.94, 94.58}	{87.50, 93.89, 84.03}
	{Hu Moments + Equivalent LBP}	{91.11, 92.92, 86.11}	{89.86, 93.47, 85.69}	{85.14, 90.97, 79.72}
	{Hu Moments + GLCM}	{81.39, 89.86, 75.56}	{85.83, 93.06, 81.53}	{91.25, 93.75, 87.64}
	{Hu Moments + Color Histogram}	{89.31, 93.75, 85.97}	{91.39, 95.42, 88.75}	{91.11, 93.47, 87.50}
	{Equivalent LBP + GLCM}	{85.42, 92.22, 81.38}	{86.81, 91.53, 81.81}	{77.08, 87.50, 71.25}
	{Equivalent LBP + Color Histogram}	{92.92, 94.58, 89.58}	{94.58, 96.39, 92.22}	{89.13, 92.22, 85.14}
	{GLCM + Color Histogram}	{90.14, 92.78, 85.83}	{92.36, 94.72, 88.89}	{88.89, 91.53, 84.86}

**Table 5:** Classification accuracy of deep learning feature-based approaches.

Methods	Descriptors	Testing Accuracy {Species, Sex, Species + Sex} (%)		
		MLP	SVM	RF
Single descriptor	{VGG16}	{85.25, 92.47, 80.30}	{95.58, 94.24, 90.58}	{86.92, 89.09, 77.22}
	{ResNet101}	{94.19, 95.38, 90.58}	{98.31, 97.80, 96.59}	{92.90, 95.35, 89.37}
	{DenseNet169}	{88.08, 93.69, 84.67}	{96.52, 97.07, 94.27}	{92.15, 90.25, 84.77}
	{MobileNetV3-L}	{85.40, 87.32, 76.19}	{89.17, 92.22, 83.54}	{80.66, 82.55, 67.35}
	{RegNetX-8GF}	{86.87, 88.13, 77.42}	{90.15, 92.27, 83.41}	{84.85, 86.74, 73.31}
	{ConvNeXt-S}	{97.20, 96.97, 95.28}	{98.74, 98.64, 97.68}	{97.45, 96.89, 94.72}
Hybrid of two descriptors	{VGG16 + ResNet101}	{95.51, 96.36, 92.70}	{95.63, 96.57, 92.75}	{86.11, 91.49, 79.62}
	{VGG16 + DenseNet169}	{93.99, 93.31, 88.03}	{91.06, 97.05, 88.61}	{89.08, 83.31, 74.42}
	{VGG16 + MobileNetV3-L}	{84.62, 82.50, 73.06}	{90.40, 95.10, 88.46}	{72.58, 78.16, 56.56}
	{VGG16 + RegNetX-8GF}	{86.74, 90.20, 78.84}	{91.59, 96.06, 88.13}	{82.50, 87.60, 71.41}
	{VGG16 + ConvNeXt-S}	{95.56, 96.06, 90.00}	{94.29, 94.24, 89.67}	{91.44, 93.91, 87.05}
	{ResNet101 + DenseNet169}	{97.05, 96.74, 94.27}	{97.55, 96.82, 94.92}	{89.37, 90.83, 81.06}
	{ResNet101 + MobileNetV3-L}	{83.71, 89.44, 75.00}	{88.08, 93.13, 83.26}	{94.90, 94.77, 90.18}
	{ResNet101 + RegNetX-8GF}	{94.37, 97.70, 92.93}	{94.12, 95.10, 89.95}	{91.67, 94.47, 86.82}
	{ResNet101 + ConvNeXt-S}	{98.08, 97.73, 95.98}	{96.76, 96.67, 94.26}	{92.02, 90.91, 84.50}
	{DenseNet169 + MobileNetV3-L}	{80.15, 89.02, 72.32}	{88.31, 91.36, 81.74}	{90.03, 84.44, 76.39}
	{DenseNet169 + RegNetX-8GF}	{94.92, 95.53, 91.06}	{88.56, 91.92, 84.62}	{76.92, 92.22, 85.76}
	{DenseNet169 + ConvNeXt-S}	{97.68, 97.60, 95.61}	{98.36, 97.88, 96.46}	{92.37, 90.73, 84.29}
	{MobileNetV3-L + RegNetX-8GF}	{83.91, 86.04, 74.60}	{92.07, 95.35, 88.51}	{76.79, 76.41, 57.30}
	{MobileNetV3-L + ConvNeXt-S}	{79.60, 89.47, 72.37}	{89.32, 91.67, 82.45}	{90.73, 95.43, 87.27}
	{RegNetX-8GF + ConvNeXt-S}	{82.70, 94.37, 78.21}	{90.68, 93.99, 85.91}	{92.22, 97.17, 90.58}

**Table 6:** Classification accuracy of fusion approaches.

Model {Descriptors + Classifiers}	Testing Accuracy {Species, Sex, Species + Sex} (%)
{HOG + ConvNeXt-S + MLP}	{99.09, 99.09, 98.40}
{HOG + ConvNeXt-S + SVM}	{98.84, 98.06, 97.25}
{Color Histogram + ConvNeXt-S +RF}	{92.60, 89.17, 83.06}
{HOG + ResNet101 + ConvNeXt-S +MLP}	{96.67, 96.06, 93.18}
{HOG + DenseNet101 + ConvNeXt-S + SVM}	{97.47, 97.17, 95.13}
{Color Histogram + RegNet-8GF + ConvNeXt-S +RF}	{73.01, 89.92, 66.84}

**Table 7:** Precision, recall, and F1-score of the selected descriptor and classifier combinations.

Approaches	Model {Descriptors + Classifiers}	Performance Measures		
		Precision	Recall	F1-Score
Traditional feature-based approaches	{HOG + MLP}	0.8854	0.8847	0.8851
	{HOG + SVM}	0.8957	0.8944	0.8951
	{Color Histogram + RF}	0.8402	0.8361	0.8382
	{HOG + Color Histogram + MLP}	0.9456	0.9444	0.9450
	{HOG + Color Histogram + SVM}	0.9468	0.9458	0.9463
	{SD-PSF + Color Histogram + RF}	0.8951	0.8931	0.8941
Deep learning feature-based approaches	{ConvNeXt-S + MLP}	0.9554	0.9528	0.9541
	{ConvNeXt-S + SVM}	0.9772	0.9767	0.9769
	{ConvNeXt-S + RF}	0.9523	0.9472	0.9497
	{ResNet101 + ConvNeXt-S + MLP}	0.9609	0.9598	0.9604
	{DenseNet169 + ConvNeXt-S + SVM}	0.9656	0.9646	0.9651
	{RegNetX-8GF + ConvNeXt-S + RF}	0.9255	0.9058	0.9156
Traditional and deep learning feature fusion approaches	{HOG + ConvNeXt-S + MLP}	0.9840	0.9838	0.9839
	{HOG + ConvNeXt-S + SVM}	0.9730	0.9725	0.9727
	{Color Histogram + ConvNeXt-S +RF}	0.8701	0.8306	0.8499
	{HOG + ResNet101 + ConvNeXt-S + MLP}	0.9379	0.9318	0.9349
	{HOG + DenseNet101 + ConvNeXt-S + SVM}	0.9522	0.9513	0.9517
	{Color Histogram + RegNet-8GF + ConvNeXt-S +RF}	0.7282	0.6684	0.6971

## Data Availability

Data will be available from the corresponding author upon reasonable request. The data are not publicly available because they are part of an ongoing study.
